# The complete chloroplast genome of a *Cymbidium Tortisepalum* (Orchidaceae) male mutant

**DOI:** 10.1080/23802359.2019.1691948

**Published:** 2019-11-18

**Authors:** Yangna Sun, Yanqiong Chen, Han Lin, Xiaokai Ma

**Affiliations:** FAFU and FAFU and UIUC-SIB Joint Center for Genomics and Biotechnology; Plant Immunity Center; The Key Laboratory of National Forestry and Grassland Administration for Orchid Conservation and Utilization (Fuzhou); College of Landscape Architecture, Fujian Agriculture and Forestry University, Fuzhou, China

**Keywords:** *Cymbidium tortisepalum*, female sterility, plastome, phylogeny, Orchidaceae

## Abstract

The chloroplast (cp) genome of natural male mutant *Cymbidium tortisepalum* ‘Guanshihe’ has been characterized using Illumina pair-end sequencing technology. The complete cp genome was 149,830 bp in length, containing a large single-copy region (LSC) of 85,131 bp and a small single-copy region (SSC) of 13,275 bp, which were separated by a pair of 25,712 bp inverted repeat regions (IRs). The genome contained 130 genes, with 111 unique genes, including 77 protein-coding genes, 30 tRNA genes, and 4 rRNA genes. The overall GC content is 37.09% with the values of the LSC, SSC, and IR regions are 34.40%, 29.63%, and 43.45%, respectively. Further, phylogenetic analysis suggested that the plastome of *C. tortisepalum* male mutant ‘Guanshihe’ is close to sequenced *C. sinense*, *C. kanran*, *C. tortisepalum*, and *C. ensifolium* plastomes.

*Cymbidium tortisepalum* Lindl., is a terrestrial orchid distributed in the southern China (Chen et al. 2009). This species contains various types of novel varieties with natural mutant flowers. Because of its high ornamental value, it is widely cultivated in southeast Asian countries. *Cymbidium tortisepalum* male mutant ‘Guanshihe’ with female sterility is one of these famous phenotypes. However, there is no information on its plastome and its relationship to other species in the genus. Here, we first reported the complete chloroplast genome of a male mutant *C. tortisepalum* ‘Guanshihe’ and its phylogenetic position in *Cymbidium* genus.

In this study, the complete chloroplast genome sequence of *C. tortisepalum* ‘Guanshihe’ was assembled and annotated. The specimen was collected from Dali, Yunnan, China (N 25°25′, E 100°00′) and then deposited in Fujian Agriculture and Forestry University (specimen voucher X.K. Ma 002). Total genomic DNA was extracted from dry leaves using a modified DNA extraction kit (Tiangen Biotech Ltd., Beijing, China) and sequenced based on the PE150 Illumina pair-end platform. The filtered reads were assembled using GetOrganelle pipe-line (https://github.com/Kinggerm/GetOrganelle) with a list of cp genomes in Orchidaceae as the reference. The assembled chloroplast genome was edited using Bandage software (Wick et al. 2015), and was then annotated using Geneious R11.15 (Biomatters Ltd., Auckland, New Zealand) (Kearse et al. 2012). The annotation result was used to draw the physical map using OGDRAW (http://ogdraw.mpimp-golm.mpg.de/) (Lohse et al. 2013).

The novel annotated complete chloroplast genome was submitted to GenBank with accession number MN497243. The complete chloroplast genome of male mutant *C. tortisepalum* ‘Guanshihe’ is 149,830 base pairs (bp) in length, containing a large single-copy (LSC) region of 85,131 bp, and a small single-copy (SSC) region of 13,275 bp, which were separated by two inverted repeat (IR) regions of 25,712 bp. The new plastome possesses total 130 genes, with 111 unique genes (including 77 protein-coding genes, 30 tRNA genes, and 4 rRNA genes). Among all of these genes, seven protein-coding genes (i.e. *ndh*B, *rpl*2, *rpl*23, *rps*12, *rps*19, *rps*7 and *ycf*2), four rRNA genes (i.e. 4.5S, 5S, 16S, and 23S rRNA), and eight tRNA genes (i.e. *trn*A-UGC, *trn*H-GUG, *trn*I-CAU, *trn*I-GAU, *trn*L-CAA, *trn*N-GUU, *trn*R-ACG, *trn*V-GAC) occur in double copies. The overall GC-content of the whole plastome is 37.09%, while the corresponding values of the LSC, SSC, and IR regions are 34.40%, 29.63%, and 43.45%, respectively.

To figure out its phylogenetic position, a ML tree was constructed based on published cp genome sequences of 12 *Cymbidium* species and two outgroup *Dendrobium* species using IQ-tree (Nguyen et al. 2015) with 1000 bootstrap replicates. The 15 sequences were aligned using Muscle (Edgar 2004). BMGE tools (Criscuolo and Gribaldo 2010) was then used to prune the aligned sequences. The ModelFinder module in IQ-tree was used to search the optimal nucleotide substitution model. Finally, the best model TVM + F+R2 was chosen to construct the phylogenetic tree according to BIC criteria using IQ-tree. Our results showed that the male mutant *C. tortisepalum* ‘Guanshihe’ is close to *C. sinense*, *C. kanran*, *C. tortisepalum*, and *C. ensifolium* with 100% bootstrap support (Figure 1). This newly reported chloroplast genome will support researchers to study the evolution of plastomes among *Cymbidium* species and their phylogenetic relationships.

**Figure 1.**
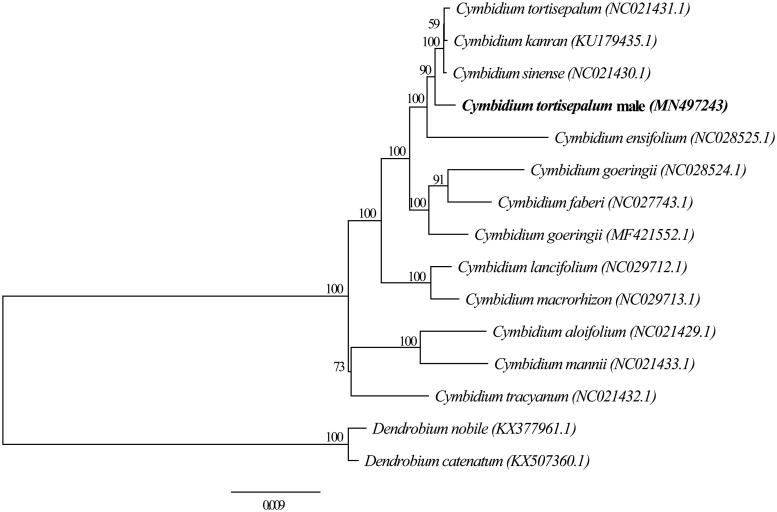
The maximum-likelihood (ML) tree based on 13 complete cp genome in *Cymbidium*, with *Dendrobium nobile* and *D. catenatum* as outgroups, boot-strap support value with 1000 replicates labeled on each node.
